# Breast cancer biomarkers predict weight loss after gastric bypass surgery

**DOI:** 10.1186/1756-0500-5-82

**Published:** 2012-01-31

**Authors:** Edward R Sauter, James E Mitchell, Beth Kliethermes, Ross D Crosby

**Affiliations:** 1 Departments of Surgery, University of North Dakota School of Medicine and Health Sciences, 501 N. Columbia Rd, Grand Forks, ND 58202, USA; 2 Clinical Neuroscience, University of North Dakota School of Medicine and Health Sciences, 501 N. Columbia Rd, Grand Forks, ND 58202, USA; 3 Neuropsychiatric Research Institute, 120 8th St. South, Fargo, ND 58103, USA

## Abstract

**Background:**

Obesity has long been associated with postmenopausal breast cancer risk and more recently with premenopausal breast cancer risk. We previously observed that nipple aspirate fluid (n) levels of prostate specific antigen (PSA) were associated with obesity. Serum (s) levels of adiponectin are lower in women with higher body mass index (BMI) and with breast cancer. We conducted a prospective study of obese women who underwent gastric bypass surgery to determine: 1) change in n- and s-adiponectin and nPSA after surgery and 2) if biomarker change is related to change in BMI. Samples (30-s, 28-n) and BMI were obtained from women 0, 3, 6 and 12 months after surgery.

**Findings:**

There was a significant increase after surgery in pre- but not postmenopausal women at all time points in s-adiponectin and at 3 and 6 months in n-adiponectin. Low n-PSA and high s-adiponectin values were highly correlated with decrease in BMI from baseline.

**Conclusions:**

Adiponectin increases locally in the breast and systemically in premenopausal women after gastric bypass. s-adiponectin in pre- and nPSA in postmenopausal women correlated with greater weight loss. This study provides preliminary evidence for biologic markers to predict weight loss after gastric bypass surgery.

## Background and Hypotheses

Thirty to 50% of postmenopausal breast cancer deaths in the U.S. have been attributed to being overweight [1]. Greater hip circumference has been linked to increased risk of premenopausal breast cancer [2]. It has been suggested that a reduction in obesity could significantly decrease breast cancer incidence [3]. In postmenopausal women without [4] and with breast cancer [5], high body mass index (BMI) is associated with increased concentrations of circulating estradiol, estrone, and testosterone. Nonetheless, BMI serves as a breast cancer risk factor independent of serum estrogen levels [6], suggesting that mechanisms other than estrogen stimulation of the breast influence breast cancer risk.

Adiponectin is an inflammatory cytokine found in and secreted by adipose cells [7]. Adiponectin has potential anticancer properties, including anti-inflammatory and insulin-sensitizing effects [8]. Serum (s) adiponectin levels have variously been associated inversely with both pre- and postmenopausal [9], or only postmenopausal [10] breast cancer risk. It has been postulated that local production of adipokines within the breast stroma surrounding epithelial cells may be directly linked to the development and growth of breast cancer and be more relevant to cancer risk than serum levels [11]. We are not aware of a published report measuring adiponectin in nipple aspirate fluid (n).

PSA, also known as kallikrein-related peptidase (KLK)3, is used to screen for prostate cancer, a disease under hormonal influence. In women, we have found that nPSA is regulated in the breast by steroid hormones [12], is inversely associated with breast cancer risk [13], and is associated with BMI in both pre- and post-menopausal women with breast cancer [14]. Serum levels of PSA are very low in women and often undetectable [15]. Our hypotheses were that: adiponectin and PSA, proteins associated with breast cancer, would change after obesity surgery, and that the change would be related to change in BMI.

## Methods

### Subjects and specimens

All subjects were female and included 25 (83.3%) Caucasians and 5 (16.7%) African Americans. Twenty were pre- and 10 postmenopausal. Median age of the group was 43.4 (range = 28-55) and median pre-surgical BMI was 47.0 (range = 36.6-60.8). BMI was similar among pre- (median = 47.0, range = 36.6-55.3) and postmenopausal (median = 45.4, range = 38.3-60.8) women. All women signed a University of Missouri Institutional Review Board approved consent prior to undergoing baseline nipple aspirate fluid and blood collection followed by Roux-en-Y gastric bypass surgery. We were able to collect nipple aspirate fluid from 28/30 (93%) women at baseline, from 25/28 (89%) women at 3 months, 25/29 (86%) women at 6 months, and 19/22 (86%) 12 months after surgery. Three women missed one appointment (two women at 3 months, one woman at 6 months). No one dropped out of the study until after the six month appointment, when eight subjects dropped out. Nipple aspirate fluid and blood procurement procedures were performed as previously described [16]. Samples were snap frozen and stored at -80°C until use. Capillary tubes containing nipple aspirate fluid were crushed to release the fluid in 200 uL of a 0.1 M sodium bicarbonate solution.

### Protein analyses

Adiponectin was analyzed using an ELISA kit from LINCO Research, Inc. (St. Charles, MO) following the manufacturer's instructions. The detection limit for the kit was 0.78 ng/ml. PSA was analyzed using a time-resolved immunofluorometric assay with a detection limit of 5 ng/L. The coefficient of variation for the adiponectin and PSA assays were < 10% within the measurement range. Total nipple aspirate fluid protein was determined using a Pierce (Rockford, IL) BCE Protein Assay Reagent kit. PSA values were controlled for total nipple aspirate fluid protein, as has been our custom in the past. Both s- and n-adiponectin were evaluated based on total fluid volume, so that values in the two body fluids could be compared.

### Statistical analysis

Summary statistics including mean, standard deviation and median were computed for the BMI and biomarkers separately by menopausal status as well as for the combined sample at pre surgery assessment and 3-, 6- and 12-months post surgery. Both means and medians are reported, means to provide standard deviations (SDs), and medians because of the limited sample size and asymmetric data, making the use of only means/SDs potentially misleading. We based our tests of significance on medians which is more conservative than on means. We feel this is appropriate with a limited sample size. Spearman's rank-order correlation coefficients were used to evaluate the association between absolute values and changes in BMI and biomarkers. Changes from baseline in BMI and biomarkers were evaluated using the Wilcoxon signed rank nonparametric test. Differences in changes in BMI and biomarkers by menopausal status were evaluated using the Mann-Whitney nonparametric test.

## Results

BMI significantly decreased over time for both pre- (*p *≤ .001) and postmenopausal (*p *< .01) women at 3, 6 and 12 months post surgery, with percent excess weight (BMI > = 25.0) slowing over time. Higher baseline BMI negatively correlated (*p *< .05) with BMI decrease (compared to baseline) at 3, 6 and 12 months, and decrease in BMI at 3 and 6 months (*p *< .05 for both) correlated with decrease at later time points.

### Adiponectin is measurable in breast nipple aspirate fluid

Since we are unaware of a prior report documenting the detection of adiponectin in nipple aspirate fluid, we first determined if adiponectin was detectable, and if detectable the concentration of the marker in this fluid relative to serum. We detected adiponectin in all serum (range: premenopausal: 2913-18402 ng/mL; postmenopausal: 4785-27179 ng/mL) and nipple aspirate fluid (range: premenopausal: 131.1-3883 ng/mL; postmenopausal: 212.2-4807 ng/mL) samples analyzed. Median n-adiponectin levels were 5.3-8.2% of s-adiponectin in pre- and 9.6-11.0% in postmenopausal women (Table 1).

**Table 1:** Biomarkers before and after gastric bypass surgery by menopausal status

Variable	Menopausal Status	Value	Months since surgery			
			
			0	3	6	12
**Nipple** **aspirate** **fluid** **(n)PSA (ng/g) ***p* value ≤ .001, ***; *p *value ≤ .01, **; *p* value ≤ .05, *, compared to baseline	Pre- menopausal	Mean	2514.0	1080.3	4942.2	1379.4
		
		Median	405.1	166.7	653.2	586.9
		
		StDev	4049.7	2406.9	11470.0	2026.8
		
		Samples	15	13	12	7
	
	Post- menopausal	Mean	1266.4	731.7	1130.5	643.2
		
		Median	200.0	109.4	575.4	399.1
		
		StDev	2831.6	1356.4	1748.7	1006.8
		
		Samples	9	8	8	8
	
	Total sample	Mean	2046.1	947.5	3417.5	986.7
	
		Median	315.7	144.3	653.2	510
	
		StDev	3626.6	2037.1	8998.0	1553.0
	
		Samples	24	21	20	15

**n-** **adiponectin** **(ng/mL) ***p* value ≤ .001, ***; *p *value ≤ .01, **; *p* value ≤ .05, *, compared to baseline	Pre- menopausal	Mean	778.7	1118.9	1164.5	1293.7
		
		Median	471.8	617.1*	808.8*	638.2
		
		StDev	746.8	961.3	1091.9	1346.2
		
		Samples	14	12	11	9
	
	Post- menopausal	Mean	1310.3	1323.1	1482.6	2056.8
		
		Median	984.2	1168.5	1006.0	1204.2
		
		StDev	992.9	903.0	1169.6	1933.4
		
		Samples	8	6	8	7
	
	Total sample	Mean	972.0	1186.9	1298.5	1627.5
		
		Median	690.9	817.1	808.8	894.8
		
		StDev	861.6	920.6	1104.7	1617.0
		
		Samples	22	18	19	16

**Serum (s)-** **adiponectin** **(ng/mL) ***p* value ≤ .001, ***; *p *value ≤ .01, **; *p* value ≤ .05,*, compared to baseline	Pre- menopausal	Mean	7245.4	9090.9	10427.7	12103.8
		
		Median	7366.0	9113.0***	9846.5***	12008.3*
		
		StDev	2706.8	2997.5	3119.2	3551.7
		
		Samples	18	17	17	8
	
	Post- menopausal	Mean	11925.6	11388.0	12370.0	13646.4
		
		Median	8900.5	10968.0	10429.5	11944.5
		
		StDev	7252.0	4829.7	4635.6	5408.2
		
		Samples	10	9	9	7
	
	Total sample	Mean	8916.9	9886.1	11100.0	12823.6
		
		Median	7406.3	9368.0**	10138.0**	11944.5**
		
		StDev	5230.6	3802.2	3740.5	4413.3
		
		Samples	28	26	26	15

### Association of nipple aspirate fluid and serum samples with BMI

nPSA did not consistently change over time (Table 1). In pre- but not postmenopausal women, n-adiponectin levels significantly increased at 3 and 6 months after surgery, and s-adiponectin increased at all post surgery time points.

**Figure 1:**
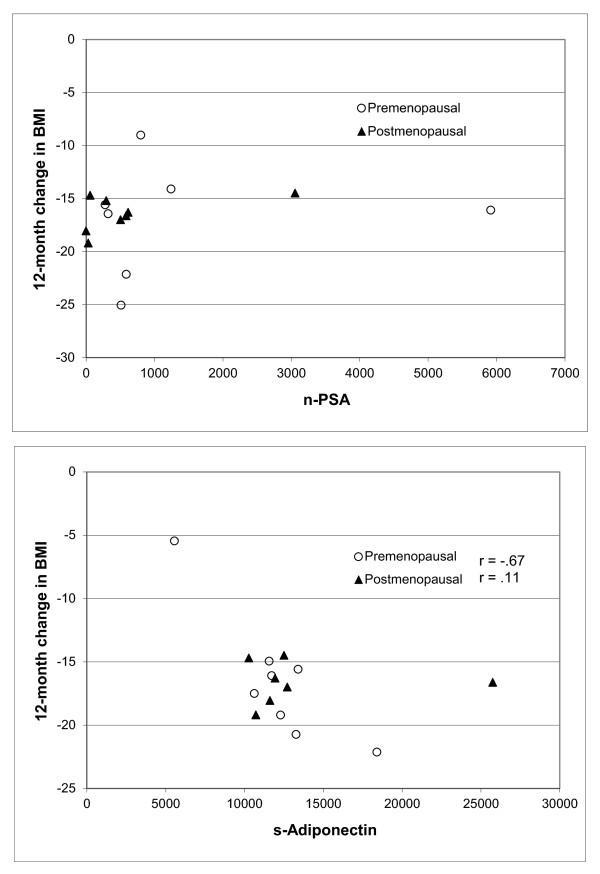
**Scatterplots illustrating change in BMI vs. A) nPSA and B) s-adiponectin 12 months after surgery with separate symbols for pre- and postmenopausal women**.

Two biomarkers, nPSA and s-adiponectin, correlated with decrease in BMI. At all time points, low nPSA correlated with BMI decrease in postmenopausal women (Figure 1A). At 6 and 12 months after surgery, high s-adiponectin (Figure 1B) correlated with BMI decrease in premenopausal women (Table 2).

**Table 2:** Spearman correlation coefficients (corr) after gastric bypass surgery^1^

			Samples/Correlation (Pvalue^1^)		
Biomarker					
**Current BMI**	**Months Since Surgery**	**0**	**3**	**6**	**12**

n-PSA	Pre	15/-.013	13/-.32	12/-.074	7/-.**43 **(.34)
	
	Post	9/-.32	8/.17	8/.21	7/.11
	
	Total	24/-.20	21/-.19	20/.026	15/-.057

n-adiponectin	Pre	14/.10	12/-.12	11/-.073	8/-.26
	
	Post	8/.12	6/.14	8/.095	7/.14
	
	Total	22/.076	18/.005	19/.053	15/-.15

s-adiponectin	Pre	18/.28	17/.**43 **(.084)	17/-.20	8/-.17
	
	Post	10/.030	9/-.083	9/.35	7/-.**50 **(.25)
	
	Total	28/.19	26/.23	26/.13	15/-.28

**BMI Δ**					

n-PSA	Pre		13/-.**45 **(.12)	12/.028	7/.32
	
	Post		8/.**66 **(.076)	8/.**55 **(.16)	8/.**60 **(.12)
	
	Total		21/.19	20/.28	15/.38

n-adiponectin	Pre		12/-.042	11/.28	8/-.33
	
	Post		6/-.**54 **(.27)	8/.21	7/.29
	
	Total		18/-.17	19/.15	15/.15

s-adiponectin	Pre		17/.007	17/-.**44 **(.076)	8/-.**67 **(.071)
	
	Post		9/-.27	9/-.15	7/.11
	
	Total		26/.091	26/-.36	15/-.39

1: *p *values are shown only for correlations ≥ 0.4. Pre: pre-menopausal; Post: post-menopausal; Total: Total sample

## Discussion

The influence of obesity on postmenopausal breast cancer risk is well established, while the association with premenpausal breast cancer risk is less certain [1]. Nonetheless, new evidence suggests that an increased hip circumference is associated with premenopausal breast cancer [2]. Multiple reports document that weight loss after gastric bypass surgery leads to a reduced risk of cancer, with the strongest effect on female obesity related tumors [17]. This risk reduction is thought to involve alterations in adipokines, sex steroid hormones and other proteins [17], but the specific proteins that influence risk and risk reduction after weight loss are for the most part unknown.

We have observed that breast specific biomarker expression is a better predictor of breast cancer risk than is expression in the circulation [15]. We demonstrate that both n- and s-adiponectin increase with a decrease in BMI, that the two are highly correlated 6 months after surgery, and that these correlations are greater in pre- than in postmenopausal women

We observed that higher levels of s-adiponectin in premenopausal women and lower levels of nPSA in postmenopausal women correlated with the amount of weight lost after surgery. Predictive markers thus far studied to determine the amount of weight that will be lost after surgery for morbid obesity include premorbid weight, exercise, and psychosocial factors [18]. We are not aware of a reliable marker present in the circulation or organ specific fluid which has been shown to predict change in BMI.

Our findings regarding adiponectin expression are consistent with the premise that weight loss lowers breast cancer risk. Higher levels of s-adiponectin [9] are found in healthy women than in women with breast cancer. Our observed increase in s-adiponectin at 12 months, as well as the inverse correlation of s-adiponectin with BMI 6 and 12 months after gastric bypass surgery, is similar to other reports [19]. Whereas median breast milk adiponectin concentrations have been reported to average 0.12% of matched serum [20], we observed that n-adiponectin averaged 5.3-8.2% of matched serum in pre- and 9.6-11.0% in postmenopausal women, suggesting higher adiponectin levels are present in the nonlactating than the lactating breast. While speculative, higher adiponectin concentrations in the nonlactating breast could lead to a greater effect of protein, which has anticancer properties [21].

There were limitations to our study, most notably a limited sample set, especially in postmenopausal women. Therefore, our findings are preliminary and require validation. Validation of our findings would suggest that noninvasive markers in both organ specific and systemic body fluids may be able to predict decrease in BMI after gastric bypass surgery, and that when assessing these markers, menopausal status should be considered.

## Abbreviations

BMI: body mass index; n-: nipple aspirate fluid; PSA: prostate-specific antigen; RYGB: Roux-en-Y gastric bypass; s-: serum.

## Competing interests

The authors declare that they have no competing interests.

## Authors' contributions

ERS designed the study, prepared all drafts of the manuscript and enrolled all participants. JEM provided scientific input in the preparation of the manuscript. BK entered all pertinent data into the database and provided this data to the statistician, RDK, who performed the statistical analysis. BK also reviewed the manuscript for accuracy, as did RDK. All authors read and approved the final manuscript.
